# Supplementary material for: A Study on Double Inputs Direct Contact and Single Output Capacitively Coupled Conductivity Detector
Source: Sensors (Basel). 2022 Apr 2;22(7):2729. doi: 10.3390/s22072729 (PMC9003331; doi:10.3390/s22072729)
Supplement: Supplementary file 1 [file sensors-22-02729-s001.zip › sensors-1616542-supplementary.pdf]

sensors

Supporting Information

A Study on Double Inputs Direct Contact and Single Output Capacitively Coupled Conductivity Detector

Shuangfei Zhang, Hongyan Yuan and Dan Xiao *

College of Chemical Engineering, Sichuan University, Chengdu 610017, China; zsfzsy2020@163.com (S.Z.); yuan_hy@scu.edu.cn (H.Y.)
* Correspondence: xiaodan@scu.edu.cn

Table S1. Different approaches for conductivity detection.

Sensor	Analtyte	Detection limit	Ref.
LIC^{4}D	K^+	0.16 mg/L	[1]
DRC^{4}D	amino acids	0.10~0.40 μM	[2]
DIC^{4}D	KCl	0.10 μM	[3]
a microfluidic platform with DC^{4}D	Tin particle	Distinguish	[4]
PC^{4}D	NaCl	10 mM	[5]
C4D based on series resonance	KCl	0~2.0 mol/L	[6]
A microchip CE-C^{4}D with integrated in-plane electrodes	K^+	18 μM	[7]
A thin-layer C^{4}D	KCl	0.5~1000 μM	[8]
DISODCD	KCl	0.001 μM	Our work

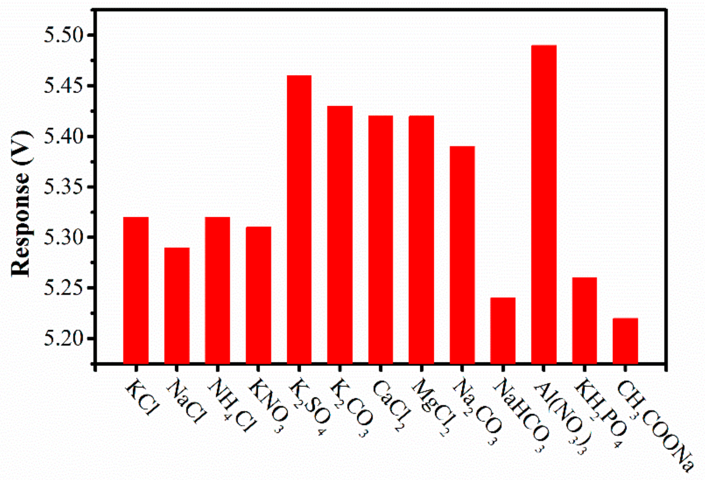

Figure S1. Response values of different electrolyte solutions at a concentration of 10 mM under DISODCD.

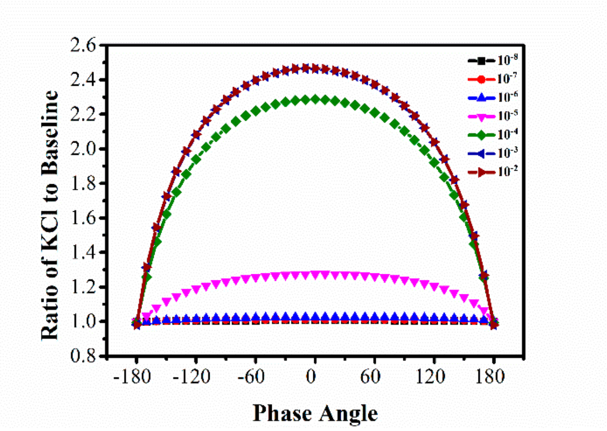

Figure S2. The ratio of the response of DISODCD at different phase 0-180°.

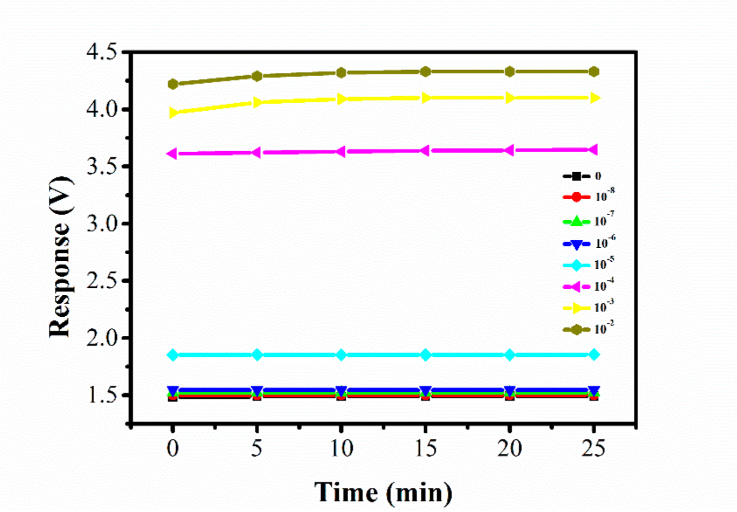

Figure S3. Response of contaminated solutions over time.

References

1. Kang, Q.; Shen, D.; Li, Q.; Hu, Q.; Dong, J.; Du, J.; Tang, B. Reduction of the Impedance of a Contactless Conductivity Detector for Microchip Capillary Electrophoresis: Compensation of the Electrode Impedance by Addition of a Series Inductance from a Piezoelectric Quartz Crystal. *Anal. Chem.* **2008**, *80*, 7826–7832. https://doi.org/10.1021/ac800380g.
2. Shen, D.; Li, Y.; Zhang, Z.; Zhang, P.; Kang, Q. Determination of amino acids by capillary electrophoresis with differential resonant contactless conductivity detector. *Talanta* **2013**, *104*, 39–43. https://doi.org/10.1016/j.talanta.2012.11.027.
3. Zheng, H.; Li, M.; Dai, J.; Wang, Z.; Li, X.; Yuan, H.; Xiao, D. Double Input Capacitively Coupled Contactless Conductivity Detector with Phase Shift. *Anal. Chem.* **2014**, *86*, 10065–10070. https://doi.org/10.1021/ac501199e
4. Do, L.Q.; Bui, T.T.; Tran, H.T.T.; Kikuchi, K.; Aoyagi, M.; Duc, T.C. Fluidic platform with embedded differential capacitively coupled contactless conductivity detector for micro-object sensing. *Int. J. Nanotechnol.* **2018**, *15*, 24–38. https://doi.org/10.1504/ijnt.2018.089543.
5. Quang, L.D.; Bui, T.T.; Hoang, A.B.; Van, T.P.; Jen, C.-P.; Duc, T.C. Development of a Passive Capacitively Coupled Contactless Conductivity Detection (PC4D) Sensor System for Fluidic Channel Analysis Toward Point-of-Care Applications. *IEEE Sens. J.* **2019**, *19*, 6371–6380. https://doi.org/10.1109/jsen.2019.2908179.
6. Huang, Z.; Jiang, W.; Zhou, X.; Wang, B.; Ji, H.; Li, H.J.S.; Chemical, A.B., A new method of capacitively coupled contactless conductivity detection based on series resonance. *Sens. Actuator B: Chem.* **2009**, 143, 239-245.
7. Lichtenberg, J.; de Rooij, N.F.; Verpoorte, E.J.E., A microchip electrophoresis system with integrated in-plane electrodes for contactless conductivity detection. *Electrophoresis.* **2002**, 23, 3769-3780.
8. Míka, J.; Opekar, F.; Coufal, P.; Štulík, K., A thin-layer contactless conductivity cell for detection in flowing liquids. *Anal Chim Acta.* **2009**, 650, 189-194.
